# Airway management in patients with deep neck infections: A retrospective analysis

**DOI:** 10.1097/MD.0000000000004125

**Published:** 2016-07-08

**Authors:** Soo Young Cho, Jae Hee Woo, Yoon Jin Kim, Eun Hee Chun, Jong In Han, Dong Yeon Kim, Hee Jung Baik, Rack Kyung Chung

**Affiliations:** Department of Anesthesiology and Pain Medicine, School of Medicine, Ewha Womans University, Seoul, Korea.

**Keywords:** airway, anesthesia, deep neck infections

## Abstract

Securing the airway in patients undergoing surgical intervention to control a deep neck infection (DNI) is challenging for anesthesiologists due to the distorted airway anatomy, limited mouth opening, tissue edema, and immobility. It is critical to assess the risk of a potential difficult airway and prepare the most appropriate airway management method.

We reviewed our anesthetic experiences managing patients with DNIs, focusing on the need for video-laryngoscope or awake fiberoptic intubation beyond a standard intubation from the anesthesiologist's perspective.

When patients had infections in the masticatory space, mouth of floor, oropharyngeal mucosal space, or laryngopharynx, their airways tended to be managed using methods requiring more effort by the anesthesiologists, and more extensive equipment preparation, compared with use of a standard laryngoscope. The degree to which the main lesion influenced the airway anatomy, especially at the level of epiglottis and aryepiglottic fold was related to the airway management method selected.

When managing the airways of patients undergoing surgery for DNIs under general anesthesia, anesthesiologists should use imaging with computed tomography to evaluate the preoperative airway status and a comprehensive understanding of radiological findings, comorbidities, and patients’ symptoms is needed.

## 1 Introduction

Deep neck infection (DNI) means infection in the potential spaces of the mucous membranes of the pharyngeal cavity and the layers of the deep neck cervical fascia.^[1]^ The management of DNI usually involves antibiotics or surgical drainage. Surgical drainage is needed when there are large abscesses, complications, or a poor response to medical management. Securing the airway in patients undergoing surgical intervention to control DNI is challenging for anesthesiologists. The distorted airway anatomy, limited mouth opening, tissue edema, and immobility make it difficult to secure the airway using intubation with direct laryngoscopy, awake blind nasal intubation, awake fiberoptic intubation, or an elective tracheostomy.^[2]^ The induction of general anesthesia may precipitate complete airway closure.^[3]^ An abscess can be ruptured, and the pus aspirated, during an attempted intubation.^[4,5]^ It is critical to assess the risk of a potential difficult airway and prepare the most appropriate airway management method.

Awake fiberoptic intubation has been recommended for managing difficult airway.^[3]^ However, it requires considerable experience and knowledge of the airway and equipment. One survey found that many anesthesiologists did not feel confident about using a fiberoptic-bronchoscope in clinical practice due to a lack of experience.^[6]^ This method can also cause complications, such as a generally unpleasant experience, hypertension, tachycardia, epistaxis, over-sedation with related hypoxemia, and airway loss. Therefore, it is important to judge when awake fiberoptic intubation is needed.^[7]^ A tracheostomy has been suggested as the gold standard for securing a difficult airway.^[5]^ However, performing a tracheostomy before inducing general anesthesia can also be challenging, because the patients usually have a distorted anatomy, which affects the location of landmarks, and dyspnea may result in difficulty in lying supine with the neck extend.^[2]^

Several studies have reported the management of DNI at various medical centers.^[8–10]^ However, few have focused on the challenging airways that anesthesiologists can face in the operating room. This study reviews our anesthetic experience over 10 years managing patients with DNI, focusing on the need for video-laryngoscope or awake fiberoptic intubation beyond a standard intubation from the anesthesiologist's perspective, and emphasizes the preoperative evaluation and appropriate management method in these patients.

## 2 Methods

The Ethics Committee of Ewha Womans University Mokdong Hospital, Seoul, Korea approved this retrospective review and analysis of patient data. This study retrospectively reviewed the records of all patients who were diagnosed with DNIs and underwent surgical drainage under general anesthesia at Ewha Woman's University Hospital between January 1995 and February 2015. Patients who were younger than 19 years were excluded. On the basis of the methods used to secure the airway, the patients were divided into 4 groups: direct Macintosh laryngoscope (Teleflex Medical Europe, Athlone, Ireland); video-laryngoscope, Pentax AWS (Pentax, Tokyo, Japan) or McGrath (Aircraft Medical, Edinburgh, UK); fiberoptic bronchoscope; or an artificial airway (intubation or tracheostomy) that had already been done before arriving in the operating room. The choice of the airway management method was at the discretion of the attending anesthesiologists considering airway physical examination, respiratory symptom, laboratory results, and radiologic evaluation. The patient demographics, general characteristics, underlying systemic diseases, DNI etiology and radiological evaluation results, and complications were analyzed.

The DNI data, including their distribution, relationship between DNI and the airway, and prevertebral soft tissue (PVST) thickness, are based on multidetector computed tomography (CT) findings. The distribution of DNI was assessed with reference to the literature,^[9,10]^ and included the masticatory, parotid, parapharyngeal, submandibular, retropharyngeal, prevertebral, carotid, and laryngopharynx, the floor of the mouth, the anterior and posterior cervical spaces, the oropharyngeal mucosal space, and the nasopharyngeal, strap, sternocleidomastoid muscles, and the subcutaneous layer of anterior neck. If 2 or more spaces were involved concurrently, the DNI was classified as multispace.

The main locations of DNIs that caused narrowing of the airway anatomy were identified. In this study, we classified the airway level into: the upper and lower halves of the oropharynx, epiglottis, and aryepiglottic fold (AEF) for the purposes of the analysis. Then, the degree of influence of the main DNIs lesion on the airway anatomy was identified from the axial CT view and classified into 5 levels: none, no involvement; mild, airway narrowing but no deviation from the midline; moderate, airway deviation but the airway cavity included the midline; severe, more airway deviation but the margin of the airway cavity bordered the patient's midline; and marked deviation, the entire airway cavity was displaced. The degrees to which the epiglottis and AEF were affected by the DNIs in each patient were each rated as none, moderate, or severe. For the epiglottis, in moderate involvement there was epiglottis swelling, but the vallecular space could be identified; while with severe involvement, the vallecula could not be distinguished from adjacent structures. For AEF, moderate involvement was indicated when there was unilateral or bilateral obliteration but 1 piriformis sinus could be identified, while with severe involvement neither piriformis sinus could be distinguished.

The PVST thickness was measured in the midline on sagittal images and determined by direct correlation with coronal reformatted images, as reported elsewhere.^[11]^ At C1 and C2, PVST thickness was measured from the craniocaudal midpoint of the C1 anterior arch and at the midpoint of the C2 vertebral body, excluding the dens to the closest point in the air column. From C3 to C6, it was measured from the midanterior vertebral body to the closest point in the air column.

## 3 Statistical analysis

SPSS for Windows software (ver. 18.0; SSS Inc., Chicago, IL) was used for the statistical analyses. Differences in the clinical data, with respect to the presence of diabetes mellitus, distribution of DNIs, method of postoperative airway management, relationship between DNIs and airway, and PVST thickness, were analyzed according to the 4 different methods used to secure the airway. In the analysis, patients who had undergone a tracheostomy and those who were already intubated on arrival in the operating room were classified into the same group. Continuous variables were analyzed by the analysis of variance or the Kruskal–Wallis test, after assessing normality and are presented as means±standard deviation or as medians with interquartile range as appropriate. Discrete variables were analyzed using the Chi-square test or Fisher exact test. The distribution of DNIs was analyzed with linear by linear association Chi-square test. PVST thickness, measured at each level according to the airway management method, was analyzed using one-way analysis of variance with Bonferroni correction applied. The PVST measurements of patients, who had been intubated or had undergone a tracheostomy, were collected, but were excluded from the group analysis because intubation has an unpredictable and significant effect on PVST thickness.^[12]^ A *P*-value less than 0.05 was considered statistically significant.

## 4 Results

During the period included in the study, 91 patients were treated surgically under general anesthesia for DNIs. Seventeen patients were younger than 19 years. Therefore, 74 patients were included in this study (55 men, 19 women; mean age, 51.27 ± 18.03 years).

Perioperative clinical details are shown in Table 1. Twenty-four patients (32.4%) had diabetes mellitus. The patients underwent surgical intervention 8.61 ± 6.29 days after the onset of symptoms related to DNIs. Before surgery, all patients were treated with antibiotics for an average of 3.16 ± 3.22 days, and 22 patients (29.7%) were treated with steroids. To evaluate the DNIs, all patients underwent CT and 1 patient also underwent magnetic resonance imaging. The CT was performed 1.42 ± 1.72 days before surgery. The patients’ airways were managed by intubation with a direct Macintosh laryngoscope in 42 patients (56.8%), a video-laryngoscope in 11 patients (14.9%), and a fiberoptic bronchoscope in 13 patients (17.6%). The Pentax AWS and McGrath were used in 3 and 8 patients, respectively. The tracheas of 4 patients (5.4%) were already intubated and 4 patients (5.4%) had a tracheostomy when they arrived in the operation room. Three anesthesiologists participated in patient management, and there was no difference in the distribution of attending anesthesiologists among the 4 groups (data not shown). No patient developed sudden airway loss during anesthesia induction.

**Table 1:**
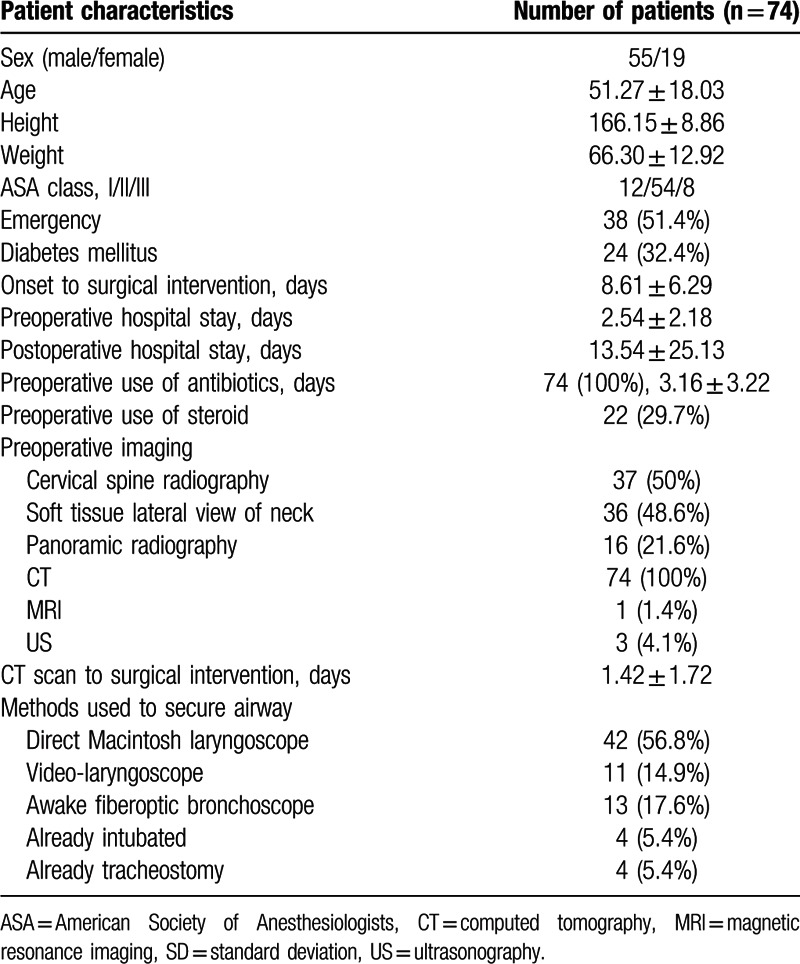
Baseline characteristics and perioperative clinical details. Values are expressed as mean (SD) or number (%).

The causes of the DNIs were identified in 50 patients (67.6%) and are shown in Table 2. Peritonsillar abscess was the most common cause (15 patients, 20.3%), followed by odontogenic infections (12 patients, 16.2%). CT was performed in all patients before surgical intervention with contrast-enhancement, except in 4 patients, of whom 3 underwent CT at another hospital and 1 had chronic kidney disease. The distribution and characteristics of the involved spaces are shown in Table 3. Of the 74 total patients, 64 (86.5%) had infections in 2 or more spaces. The percentage of patients in whom multiple spaces were involved concurrently did not differ significantly among airway management methods; the side involved (right, left, or both) also did not differ by management method. The involved spaces are shown in Table 3, and the laryngopharynx (63.9%), oropharyngeal mucosal space (50%), and mandibular space (45.8%) were involved most frequently. Depending on the method used for securing the airway, there was a significant increasing trend (*P* for trend < 0.05, Table 3) in the number of patients with infections in the masticatory space, mouth of floor, oropharyngeal mucosal space, and laryngopharynx. The 8 patients who were already intubated or had a tracheostomy all had infections in the laryngopharynx and 7 (87.5%) had infections in the oropharyngeal mucosal space. The methods used for postoperative airway control differed significantly among the groups. Only 1 patient in the laryngoscopy group was transferred to the intensive care unit while still intubated, and only 60%, 30.8%, and 0% of the patients treated using a video-laryngoscope, awake fiberoptic bronchoscope, or who already had an artificial airway, respectively, could be extubated at the conclusion of surgery. There were no patients who experienced a reintubation or an unplanned extubation.

**Table 2:**
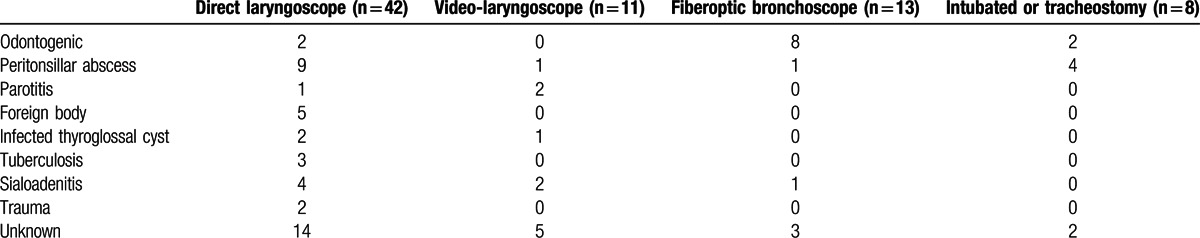
Etiology of deep neck infection. Values are expressed as number.

**Table 3:**
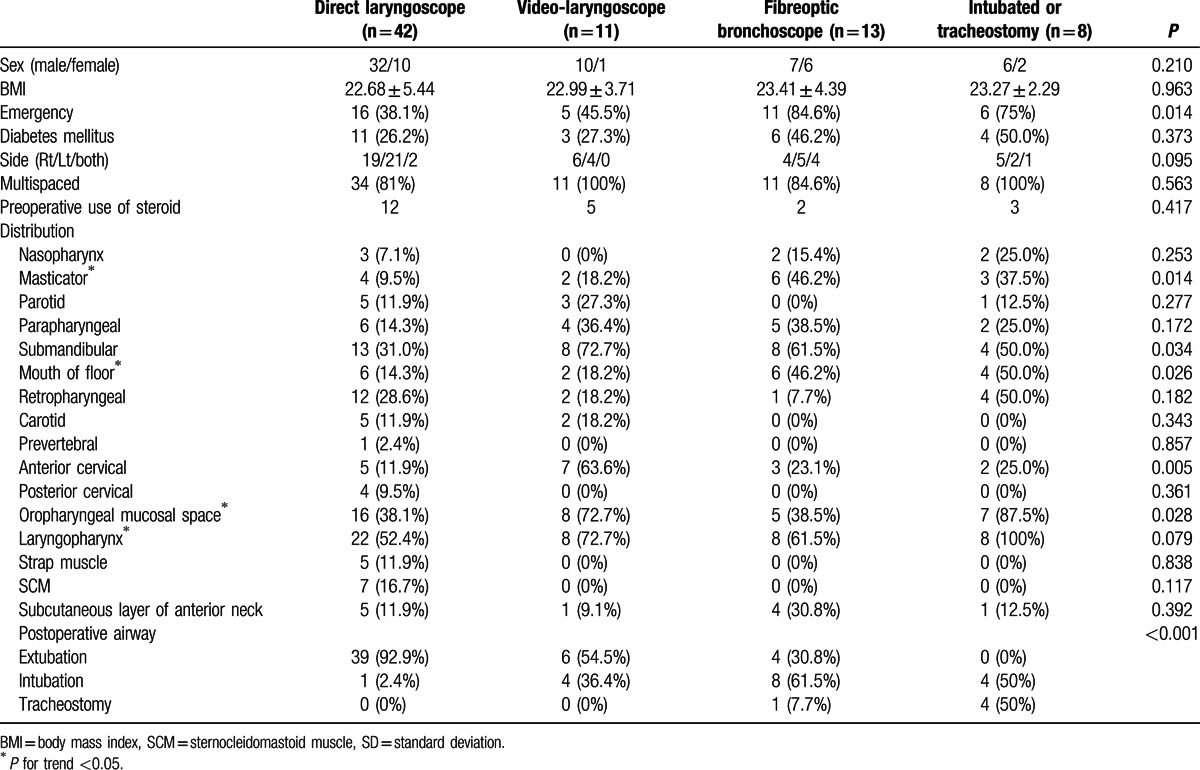
Clinical data of patients undergoing general anesthesia due to surgical control of deep neck infection. Values are expressed as mean (SD) or number (%).

Of the patients intubated with a laryngoscope, 14 (33.3%) had a main DNI lesion that was not related to the airway structure, whereas the DNI influenced the airway in almost all of the other patients. These lesions most frequently affected the airway at the level of the epiglottis or AEF. The degree of epiglottis and AEF involvement by infection differed significantly among the 4 groups. In the laryngoscope group, 27 (64.3%) and 21 (50%) patients had intact anatomy of the epiglottis and AEF, respectively, whereas these structures were much more frequently involved with the infections in the other patient groups, as shown in Table 4. The presence and number of abscesses did not differ among groups.

**Table 4:**
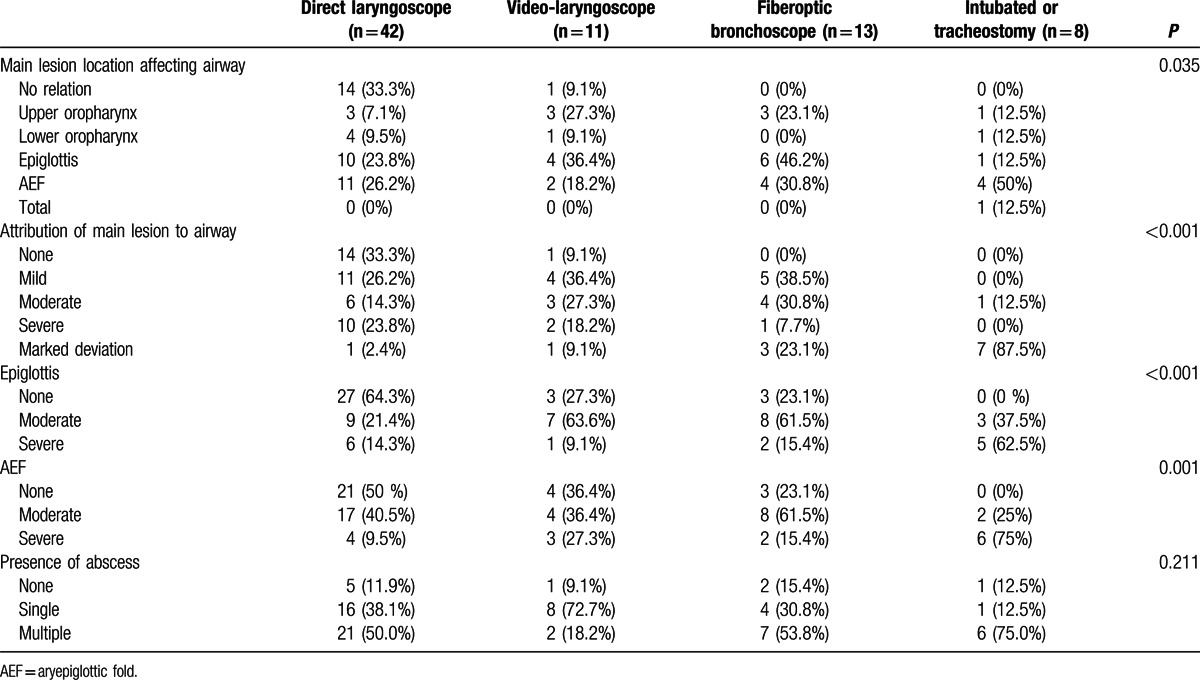
Computed tomography scans of the head and neck. Values are expressed as number (%).

Table 5 shows the PVST thickness measured at levels C1 to C6. After applying the Bonferroni correction for the comparison of three groups (excluding patients with a tracheostomy or an already intubated trachea), there was no significant difference in the PVST thickness.

**Table 5:**
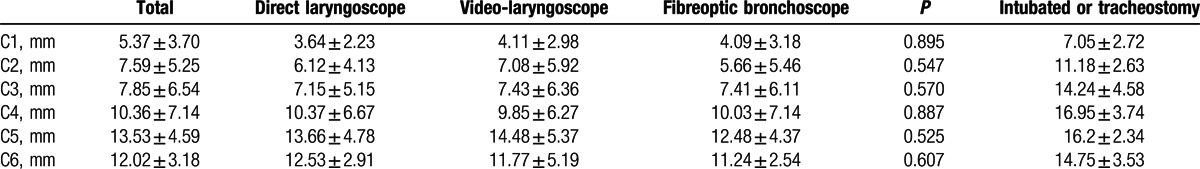
Prevertebral soft tissue thickness of C1 to C6. Values are expressed as mean (standard deviation [SD]).

Table 6 shows the complications occurring in our patients. One patient died from worsening pneumonia 2 weeks postoperatively. He was a 71-year-old man, with underlying alcoholic liver cirrhosis and interstitial pneumonia, who underwent surgery because his peritonsillar abscess in the right palatine tonsil increased despite 5 days of antibiotics. Multiple abscesses were also identified in the parapharyngeal, right submandibular, and anterior cervical spaces, and there was fluid collection in the retropharyngeal space. His epiglottis and AEF were swollen, although the vallecula and piriformis sinus on both sides could be distinguished from adjacent tissue. On arrival in the operating room, he had mild respiratory distress and his trachea was intubated using a laryngoscope. He was the 1 patient in the laryngoscope group who was transferred to the intensive care unit while still intubated because arterial blood gas analysis on emergence from anesthesia showed CO_2_ retention, and he also had underlying lung disease.

**Table 6:**
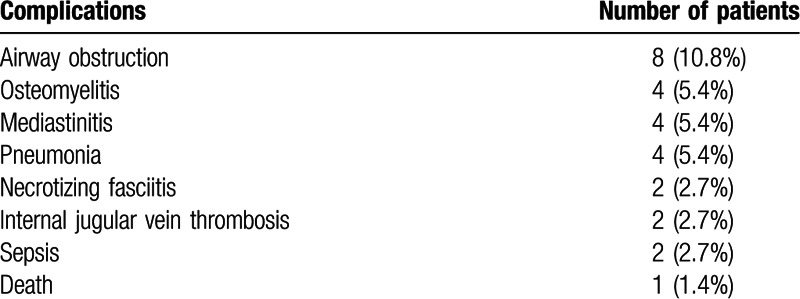
Complications of deep neck infection. Values are expressed as number (%).

## 5 Discussion

This study evaluated the perioperative patient characteristics and preoperative imaging results according to the airway management method. When patients had infections in the masticatory space, mouth of floor, oropharyngeal mucosal space, or laryngopharynx, their airways tended to be managed using methods requiring more effort by the anesthesiologists, and more extensive equipment preparation, compared with use of a standard laryngoscope. The degree to which the main DNI lesion influenced the airway anatomy especially at the level of epiglottis and AEF was related to the airway management method selected.

Contrast-enhanced CT has 64% to 100% sensitivity for identifying the extent and characteristics of DNIs.^[1,13]^ Although anesthesiologists might not be trained in the interpretation of CT findings, this study indicates that it is important to assess risk using preoperative CT findings, with respect, for example, to the distribution of infection and relationship with the airway, when treating patients with DNIs surgically. It has been reported that radiological findings of cellulitis indicate a better prognosis, whereas abscesses indicate more aggressive clinical progress. In our series, the choice of airway management method was not related to the presence of abscess, DNI character (cellulitis versus abscess), or number of spaces involved. Even if an infection involves multiple spaces and is large, it may still not complicate airway management if it is located in an area such as the sternocleidomastoid muscle or posterior cervical space, or involves anterior neck cellulitis, which might appear serious externally. If an infection progresses aggressively, the preoperative CT findings may underestimate the risk of a difficult airway. In our patients, surgery was performed an average of 1.42 ± 1.72 days after the last CT study, which might have influenced our analysis of the CT findings according to the airway management method.

PVST thickness measured on a lateral cervical X-ray or CT image can be used to identify soft tissue abnormalities, such as prevertebral abscesses, edema, hematomas, or tumors.^[11,14]^ According to Rojas et al,^[11]^ the upper limit of normal PVST thickness measured using multidetector CT in the neutral position in adults is 8.5, 6, and 7 mm at C3, C4, and C5, respectively. In our patients, the average PVST thickness at C2 and C3 exceeded the upper limits of normal. Soft tissue abnormalities are more commonly detected at C2 and C3 than at lower levels because of the relatively greater laxity of the PVST.^[15]^ The PVST thickness at the C4 and C5 levels shows great variation due to the variable locations of the esophagus and larynx, and according to the phase of swallowing; therefore, it is difficult to determine the upper normal limits of PVST at C4 and C5.^[11]^ We found no difference in PVST thickness among the 3 groups, excluding the 8 patients with a tracheostomy or who were already intubated. Some points need to be considered when interpreting our results, as there were only 19 patients (25.7%) with DNIs of the retropharyngeal space and they were not distributed evenly among the groups. In addition, because this is a retrospective analysis, the patient's position could not be controlled, which could have influenced the PVST thickness.

One retrospective analysis described the experience of awake fiberoptic intubation in 24 patients with DNIs undergoing surgical intervention under general anesthesia between 1978 and 1998.^[7]^ It was concluded that, in experienced hands, awake fiberoptic intubation can be performed safely as the first choice to control the airway in adults with DNIs, and that tracheostomy is recommended if fiberoptic intubation is not available or has failed. As DNI patients are prone to having a distorted airway, awake fiberoptic intubation would be challenging, particularly for inexperienced anesthesiologists. Failure of awake fiberoptic intubation is due more often to a lack of experience than to the difficulty of the airway itself. Airway irritation and bronchospasm caused by inexperience with fiber-bronchoscopy is a critical factor in sudden airway loss.^[16,17]^ Recently, the video-laryngoscope was introduced into clinical practice; this device benefits from being easy to use.^[18]^ Recent studies indicate that the video-laryngoscope has advantages over the Macintosh for difficult airways, and in obese patients and those with cervical spine mobilization. In our institution, video-laryngoscopy has been used for patients with difficult airways and cervical spine injuries since it was introduced in 2009. In Rosenstock randomised trial,^[19]^ awake video-laryngoscopic intubation was suggested as an alternative to awake fiberoptic intubation for difficult airways. In this study, we did not use a video-laryngoscope for awake intubation. However, in a context similar to that of Rosenstock study, in some of our patients, airway patency was checked using gentle video-laryngoscopy under topical anesthesia before inducing general anesthesia. Because even minimal sedation can collapse the airway, we believe that general anesthesia can be induced after a video-laryngoscope assessment under topical anesthesia, if it is not certain that the patient's airway can be intubated and the patient is cooperative. However, it should still be noted that even brief gentle laryngoscopy and topical anesthesia can cause laryngospasm and subsequent airway collapse.^[20,21]^

Our study had several limitations. First, it used a retrospective, single-center design. Second, we compared patient characteristics and radiological findings according to the method used to secure the airway; however, it is possible that the method we used was not the best option for the patient. Awake fiberoptic intubation was performed not only when laryngoscope or video-laryngoscope failed, so the decision regarding use might have differed depending on the attending anesthesiologist. However, all of the patients were assessed by experienced senior anesthesiologists, following discussions that considered the symptoms of airway compromise. It was not the purpose of this study to suggest specific characteristics and parameters for deciding the optimal airway management method. Further studies are warranted to evaluate which radiological parameters can predict the occurrence of a “cannot be ventilated and cannot be intubated” situation, and which airway management method is optimal for specific patients with a DNI. Our results provide a basis for further well-designed prospective studies of this population. Last, some conclusions regarding the etiology of infection, characteristics of infections in certain spaces, and complications could not be drawn due to the small numbers of patients.

## 6 Conclusion

The location of an infection and its effects on normal airway anatomy, rather than its size alone, are critical when preparing to secure the airway in DNI patients. Although they are not familiar with CT evaluation, anesthesiologists should use CT to evaluate the preoperative airway status rather than leaving the evaluation to the surgeon alone. A comprehensive understanding of radiological findings, comorbidities, and patients’ symptoms is needed when managing the airways of patients undergoing surgery for DNIs under general anesthesia.

## References

[1] Wang B Gao BL Xu GP Images of deep neck space infection and the clinical significance. *Acta Radiol* 2014; 55:945–951. 24249813 10.1177/0284185113509093

[2] Karkos PD Leong SC Beer H Challenging airways in deep neck space infections. *Am J Otolaryngol* 2007; 28:415–418. 17980775 10.1016/j.amjoto.2006.10.012

[3] Varghese BT Balakrishnan M Kuriakose R Fibre-optic intubation in oncological head and neck emergencies. *J Laryngol Otol* 2005; 119:634–638. 16102221 10.1258/0022215054516160

[4] Neff SP Merry AF Anderson B Airway management in Ludwig's angina. *Anaesth Intensive Care* 1999; 27:659–661. 10631426 10.1177/0310057X9902700323

[5] Irani BS Martin-Hirsch D Lannigan F Infection of the neck spaces: a present day complication. *J Laryngol Otol* 1992; 106:455–458. 1613380 10.1017/s0022215100119826

[6] Allan AG Reluctance of anaesthetists to perform awake intubation. *Anaesthesia* 2004; 59:413. 10.1111/j.1365-2044.2004.03729.x 15023129

[7] Ovassapian A Tuncbilek M Weitzel EK Airway management in adult patients with deep neck infections: a case series and review of the literature. *Anesth Analg* 2005; 100:585–589. 15673898 10.1213/01.ANE.0000141526.32741.CF

[8] Bakir S Tanriverdi MH Gun R Deep neck space infections: a retrospective review of 173 cases. *Am J Otolaryngol* 2012; 33:56–63. 21414684 10.1016/j.amjoto.2011.01.003

[9] Huang TT Liu TC Chen PR Deep neck infection: analysis of 185 cases. *Head Neck* 2004; 26:854–860. 15390207 10.1002/hed.20014

[10] Lee YQ Kanagalingam J Deep neck abscesses: the Singapore experience. *Eur Arch Otorhinolaryngol* 2011; 268:609–614. 20857130 10.1007/s00405-010-1387-8

[11] Rojas CA Vermess D Bertozzi JC Normal thickness and appearance of the prevertebral soft tissues on multidetector CT. *AJNR Am J Neuroradiol* 2009; 30:136–141. 19001541 10.3174/ajnr.A1307 PMC7051716

[12] Moch AL Schweitzer ME Parker L Prevertebral soft tissue swelling following trauma: usefulness following tube placement. *Skeletal Radiol* 2000; 29:340–345. 10929416 10.1007/s002560000197

[13] Santos Gorjon P Blanco Perez P Morales Martin AC Deep neck infection. Review of 286 cases. *Acta Otorrinolaringol Esp* 2012; 63:31–41. 21820639 10.1016/j.otorri.2011.06.002

[14] Chen MY Bohrer SP Radiographic measurement of prevertebral soft tissue thickness on lateral radiographs of the neck. *Skeletal Radiol* 1999; 28:444–446. 10486012 10.1007/s002560050543

[15] Penning L Prevertebral hematoma in cervical spine injury: incidence and etiologic significance. *AJR Am J Roentgenol* 1981; 136:553–561. 6781295 10.2214/ajr.136.3.553

[16] McGuire G el-Beheiry H Complete upper airway obstruction during awake fibreoptic intubation in patients with unstable cervical spine fractures. *Can J Anaesth* 1999; 46:176–178. 10083999 10.1007/BF03012553

[17] Shaw IC Welchew EA Harrison BJ Complete airway obstruction during awake fibreoptic intubation. *Anaesthesia* 1997; 52:582–585. 9203888 10.1111/j.1365-2044.1997.155-az0155.x

[18] Niforopoulou P Pantazopoulos I Demestiha T Video-laryngoscopes in the adult airway management: a topical review of the literature. *Acta Anaesthesiol Scand* 2010; 54:1050–1061. 20887406 10.1111/j.1399-6576.2010.02285.x

[19] Rosenstock CV Thogersen B Afshari A Awake fiberoptic or awake video laryngoscopic tracheal intubation in patients with anticipated difficult airway management: a randomized clinical trial. *Anesthesiology* 2012; 116:1210–1216. 22487805 10.1097/ALN.0b013e318254d085

[20] Hamilton ND Hegarty M Calder A Does topical lidocaine before tracheal intubation attenuate airway responses in children? An observational audit. *Paediatr Anaesth* 2012; 22:345–350. 22211867 10.1111/j.1460-9592.2011.03772.x

[21] Arslan IB Kose I Ciger E Does topical anesthesia using aerosolized lidocaine inhibit the superior laryngeal nerve reflex? *Otolaryngol Head Neck Surg* 2013; 149:466–472. 23818488 10.1177/0194599813495372

