# Airway management in patients with deep neck infections: A retrospective analysis: Erratum

**DOI:** 10.1097/01.md.0000504793.45336.c2

**Published:** 2016-10-21

In the article “Airway management in patients with deep neck infections: A retrospective analysis”,^[1]^ which appeared in Volume 95, Issue 27 of *Medicine*, Dr. Youn Jin Kim's name was originally misspelled. The article has since been corrected online.
